# Big Potential From Silicon-Based Porous Nanomaterials: In Field of Energy Storage and Sensors

**DOI:** 10.3389/fchem.2018.00539

**Published:** 2018-11-08

**Authors:** Rana Zafar Abbas Manj, Xinqi Chen, Waheed Ur Rehman, Guanjia Zhu, Wei Luo, Jianping Yang

**Affiliations:** ^1^State Key Laboratory for Modification of Chemical Fibers and Polymer Materials, College of Materials Science and Engineering, Donghua University, Shanghai, China; ^2^School of Physics and Mechanical and Electrical Engineering, Hubei University of Education, Wuhan, China; ^3^Institute of Functional Materials, Donghua University, Shanghai, China

**Keywords:** porous structures, silicon nanomaterials, core shell, bioapplication, lithium ion battery

## Abstract

Silicon nanoparticles (SiNPs) are the promising materials in the various applications due to their unique properties like large surface area, biocompatibility, stability, excellent optical and electrical properties. Surface, optical and electrical properties are highly dependent on particle size, doping of different materials and so on. Porous structures in silicon nanomaterials not only improve the specific surface area, adsorption, and photoluminescence efficiency but also provide numbers of voids as well as the high surface to volume ratio and enhance the adsorption ability. In this review, we focus on the significance of porous silicon/mesoporous silicon nanoparticles (pSiNPs/mSiNPs) in the applications of energy storage, sensors and bioscience. Silicon as anode material in the lithium-ion batteries (LIBs) faces a huge change in volume during charging/discharging which leads to cracking, electrical contact loss and unstable solid electrolyte interphase. To overcome challenges of Si anode in the LIBs, mSiNPs are the promising candidates with different structures and coating of different materials to enhance electrochemical properties. On the basis of optical properties with tunable wavelength, pSiNPs are catching good results in biosensors and gas sensors. The mSiNPs with different structures and modified surfaces are playing an important role in the detection of biomarkers, drug delivery and diagnosis of cancer and tumors.

## Introduction of silicon-based nanostructures

Silicon nanoparticles (SiNPs) have been remained a material of great interest with versatile and promising applications compared to the bulk material due to their physical and chemical properties (Dinh et al., 1996; Shiohara et al., 2009). SiNPs render a range of properties and series of functionalization (chemical and biological species), non-toxicity, biocompatibility and solubility in physiological fluid (Kang et al., 2009; Park et al., 2009; Wang et al., 2012). Electrical properties of silicon rely on temperature which is a conductor at room temperature. Electronic conductivity of SiNPs can be enhanced by doping of 3rd and 4th group elements of periodic table, functionalization and particle size (Anderson and Spear, 1977; Arora et al., 1982; Van Buuren et al., 1998; Veinot, 2006; Sivakov et al., 2009). SiNPs with different dyes also exhibit good photoluminescence (PL), fluorescent properties, tunable wavelength of excitation and emission, excellent photo and chemical stability (Chen et al., 2001; English et al., 2002; Shen et al., 2010; Intartaglia et al., 2012). Optical properties of SiNPs strongly depend upon quantum confinement effect which varies with changes in particle size, concentration, and functionalization (Trwoga et al., 1998; Dancil et al., 1999; Ledoux et al., 2002). Due to these unique properties, SiNPs show numbers of applications in different fields like drug carriers, bioimaging, gas sensors, solar cells, electronics and energy storage devices.

In order to improve the performance in different applications, the structure of silicon has modified into the porous structure. On the basis of pores size, porous silicon is classified into microporous (pore diameter <2 nm), mesoporous (pore diameter 2–50 nm) and macro porous (pore diameter >50 nm). Because of alternation in the structure of silicon, a tremendous variation happen in optical properties of SiNPs which can attribute to the reduction in refractive index and enhancement in PL efficiency as compare to silicon at room temperature (Bsiesy et al., 1991; Astrova and Tolmachev, 2000; Chao, 2011; Min-Dianey et al., 2018). Surface properties also affected by varying the structure of silicon, the specific surface area per unit volume increase owing to pores in structure. Mesoporous structure of silicon improves surface to volume ratio, physical adsorption and electrical resistivity of silicon due to large void spaces (Karlsson et al., 2004; Hajji et al., 2006; Lasave et al., 2013; Azadeh et al., 2018). On behalf of these structural base properties, porous silicon nanomaterials have high potential to resolve challenges in different fields i.e. energy storage devices, sensors and biomedical applications (Yang et al., 2014a, 2016; Wang et al., 2015).

## Applications of silicon-based porous nanomaterials

### Lithium-ion batteries

**Figure 1:**
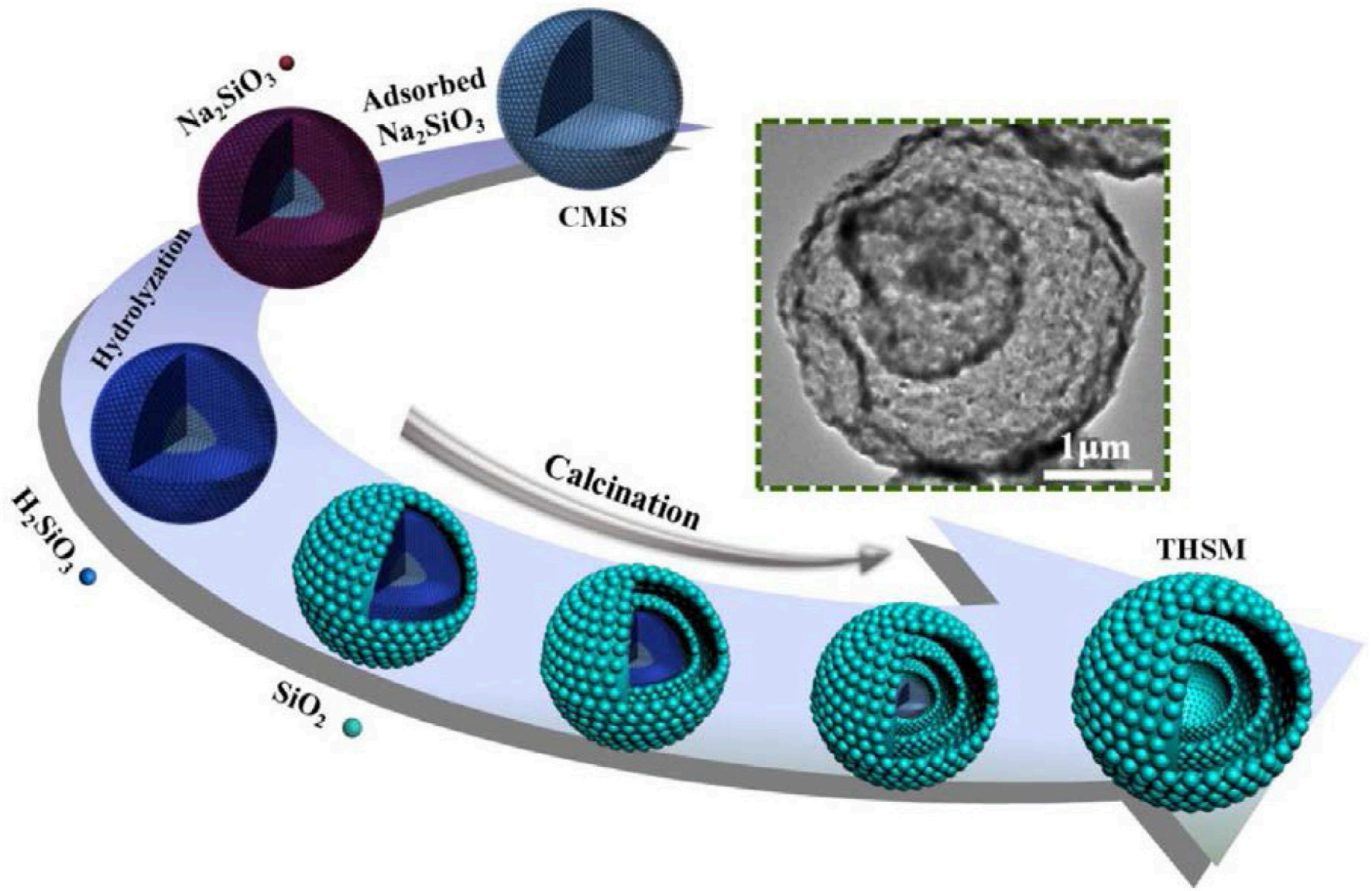
Schematic diagram of preparation of multi-shell hollow silica sphere (Ma et al., 2017b). Copyright © 2017 Elsevier B.V. All rights reserved.

**Figure 2:**
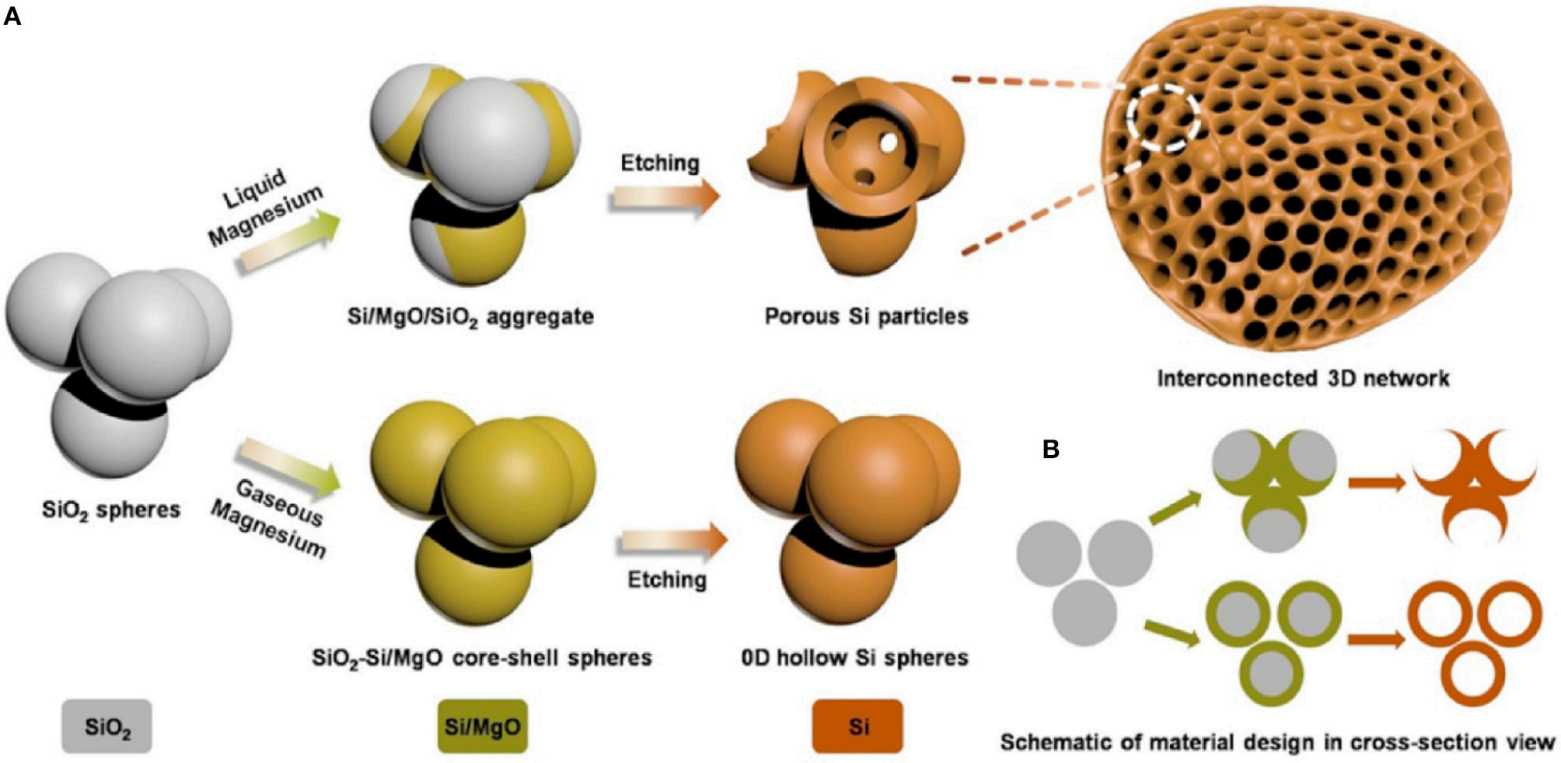
Schematic diagram of a process of synthesis mesoporous silicon, in **(A,B)**, three-dimensional (3D) view and two-dimensional (2D) cross-sectional view (Wu et al., 2016). Copyright © 2016 Elsevier B.V. All rights reserved.

Si as anode material has very high theoretical specific capacity 4,200 mA h g^−1^ as compare to graphite (Goodenough and Kim, 2009). In spite of high specific capacity of Si, Si as anode material has a major problem of volume expansion (up to 400%) during charging/discharging which lead to cracking of anode, electrical contact loss, unstable solid electrolyte interphase (SEI) film and finally fast fading of capacity (Yang et al., 2002; Jung et al., 2003; Baranchugov et al., 2007). Besides, the low intrinsic electrical conductivity (1.56 × 10^−3^ S m^−1^) and lithium diffusivity of Si also limits its electrochemical performance. It has been noted that micro and nanostructure of silicon have good electrochemical performance as compare to bulk silicon as anode material (Ryu et al., 2004; Kim et al., 2008). The volume expansion of silicon anode material has been controlled by introducing nanostructure of silicon as Si micro/nanostructures, nanowires, hollow-structured and porous silicon (Li et al., 2000; Huang and Zhu, 2010; Xu et al., 2011; Wen et al., 2013; Cho et al., 2017; Kim et al., 2017). Spherical nano-particles of silicon having diameter of 150–200 nm was used as bulk silicon anode material which exhibited capacity higher than 500 mA h g^−1^ at current 0.2 A g^−1^ up to 100 cycles (Liang et al., 2018). To further enhance the electrochemical properties, silicon nanowires (SiNWs) on stainless steel was prepared by CVD process. SiNWs as anode material delivered great area capacity of 7.1 cm with retention 60 % at current rate C/50 and good rate performance (Leveau et al., 2016). To accommodate volume expansion of Si anode and cycling stability, the structure of silicon was modified to the spherical hollow structure of silicon as shown in Figure 1. The multi-shell hollow silica microsphere (MHSM) was prepared by the sacrificial template process. MHSM as anode material delivered high capacity 750 mA h g^−1^ at current 0.1 A g^−1^ after 500 cycles. MHSM exhibited excellent cycling stability because of a porous structure which provides the easy reaction among the lithium ion and anode material and reduced the reaction path (Ma et al., 2017b). Further, three-dimensional (3D) macroporous silicon was synthesized by magnesiothermic reduction to enhance structure stability, capacity and cycle life of Si anode as shown in Figure 2. 3D porous silicon as anode material showed high capacity with excellent retention and cycle life (1,058 mA h g^−1^ with current rate 2 A g^−1^ after 800 cycles) (Wu et al., 2016). The structural modification in silicon anode material provided a significant improvement in electrochemical performance as compared to bulk silicon anode. The large area of nano-structure and greater voids of porous structure provided a large surface to the diffusivity of lithium ions and controlled the pulverization and compensated the electrical contact loss.

#### Silicon nanostructures with binders in LIBs

**Figure 3:**
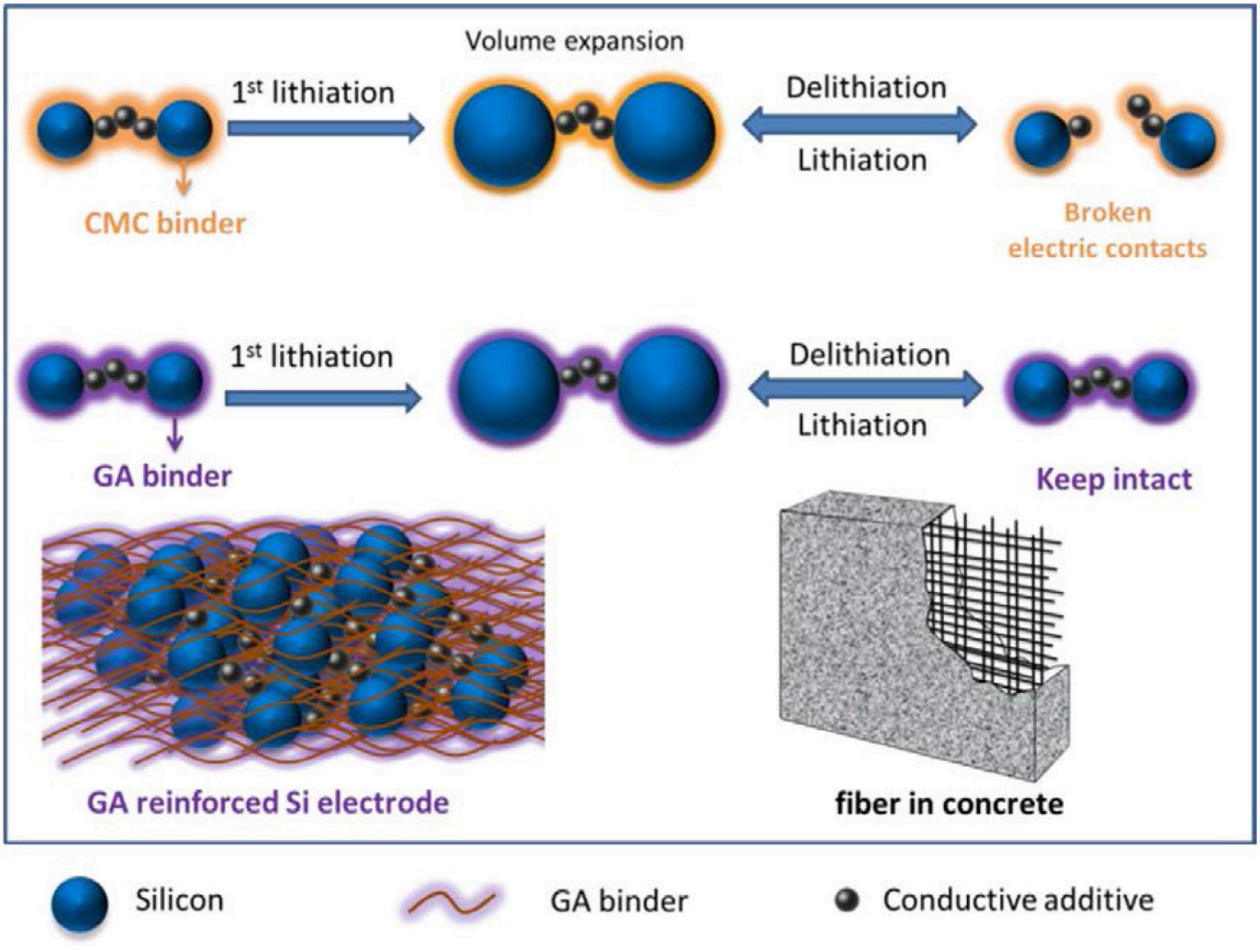
Schematic diagram to elaborate the working of CMC and GA binder during volume change in charging/discharging (Ling et al., 2015). Copyright © 2014 Elsevier Ltd. All rights reserved.

The nano-structural silicon enhanced the electrochemical performance to some extent, but a modification in silicon anode was still required. Further electrical contact loss in silicon anode due to pulverization was controlled by adding conductive additives and polymer binders in Si anode (Priyono et al., 2017; Teng et al., 2018). Conductive additives provided an electronic path in charging/discharging but after some cycles failed to contact silicon particles because conductive additives could not provide binding force Si particles (Beattie et al., 2008; Renganathan et al., 2010). To overcome these issues, numbers of conductive binders were used to improve electronic conductivity and stability of Si anode for long cycle life (Lin et al., 2016; Sarode et al., 2018). By taking the advantages of self-healing ability of a material, a conductive binder was prepared by use of ureidopyrimidinone (UPy) and polyethylene glycol (PEG). By use UPy-PEG-UPy as a binder with SiNPs in anode material excellent electrochemical performance was achieved. SiNPs anode with UPy-PEG-UPy binder delivered high capacity 1,454 mA h g^−1^ over 400 cycles with the decay of 0.04 % per cycle. The self-healing ability of UPy-PEG-UPy binder maintained the good integrity of silicon anode as compared to the traditional binder (Yang et al., 2018). Electrical contact loss in Si anode has been tried to control by the chemically bonded conductive binder. The aqueous hybrid gel prepared with sodium carboxymethyl cellulose by cross-linker sodium borate and used with silicon as an anode material. Hybrid gel covalently bonded with silicon particles and also acted as a buffer for silicon particles. Si anode with gel exhibited good capacity and cycle life of 1211.5 mA h g^−1^ after 600 cycles with coulombic efficiency 88.95 % (Zhang et al., 2018). Furthermore, to enhance the electrochemical properties and stability of Si anode, a naturally derived gum Arabic (GA) polymer (as fiber-reinforced concrete) was used to control cracking of Si anode during charging/discharging as shown in Figure 3. Si anode with GA polymer binder delivered high capacity as compare to CMC binder 2,000 mA h g^−1^ at current rate 1C after 500 cycles and excellent cycle life with capacity 1,000 mA h g^−1^ at rate 1C over 1,000 cycles. Glycoprotein chain in GA provided good mechanical properties which behaved like a fiber in concrete and polysaccharide provided binding force due to the presence of hydroxyl groups (Ling et al., 2015). Conductive binder polymers have significantly enhanced the electrochemical properties of Si anode material and its structural integrity.

#### Coated-silicon nanostructures in LIBs

**Figure 4:**
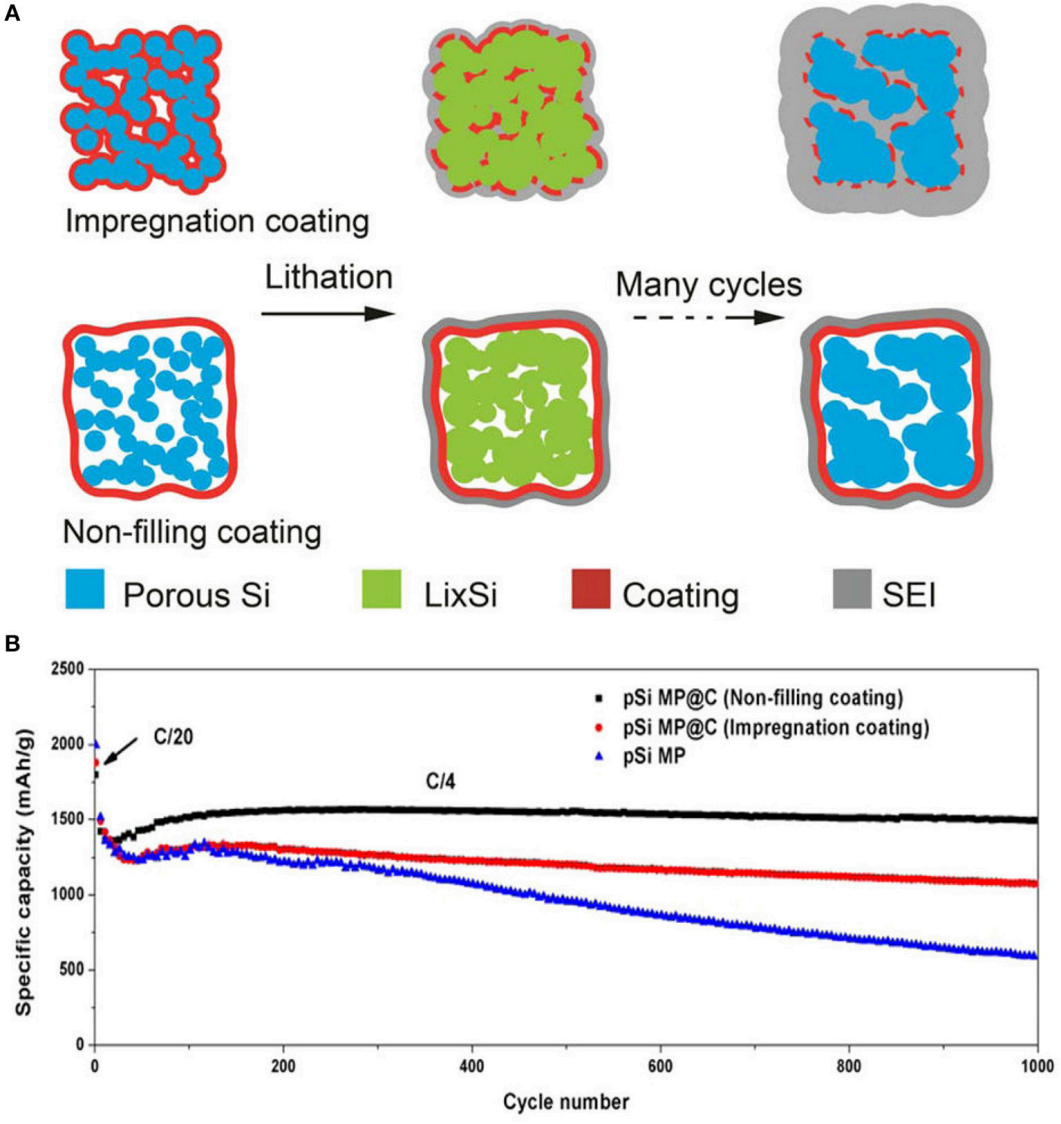
**(A)** Schematic diagram of the coating process, the evolution of SEI film of impregnation and non-filling coating during cycling. **(B)** Analysis of specific capacity of non-filling, impregnation coated pSiMPs over 1,000 cycles. Reproduced with permission (Lu et al., 2015). Copyright © 2015, American Chemical Society.

**Figure 5:**
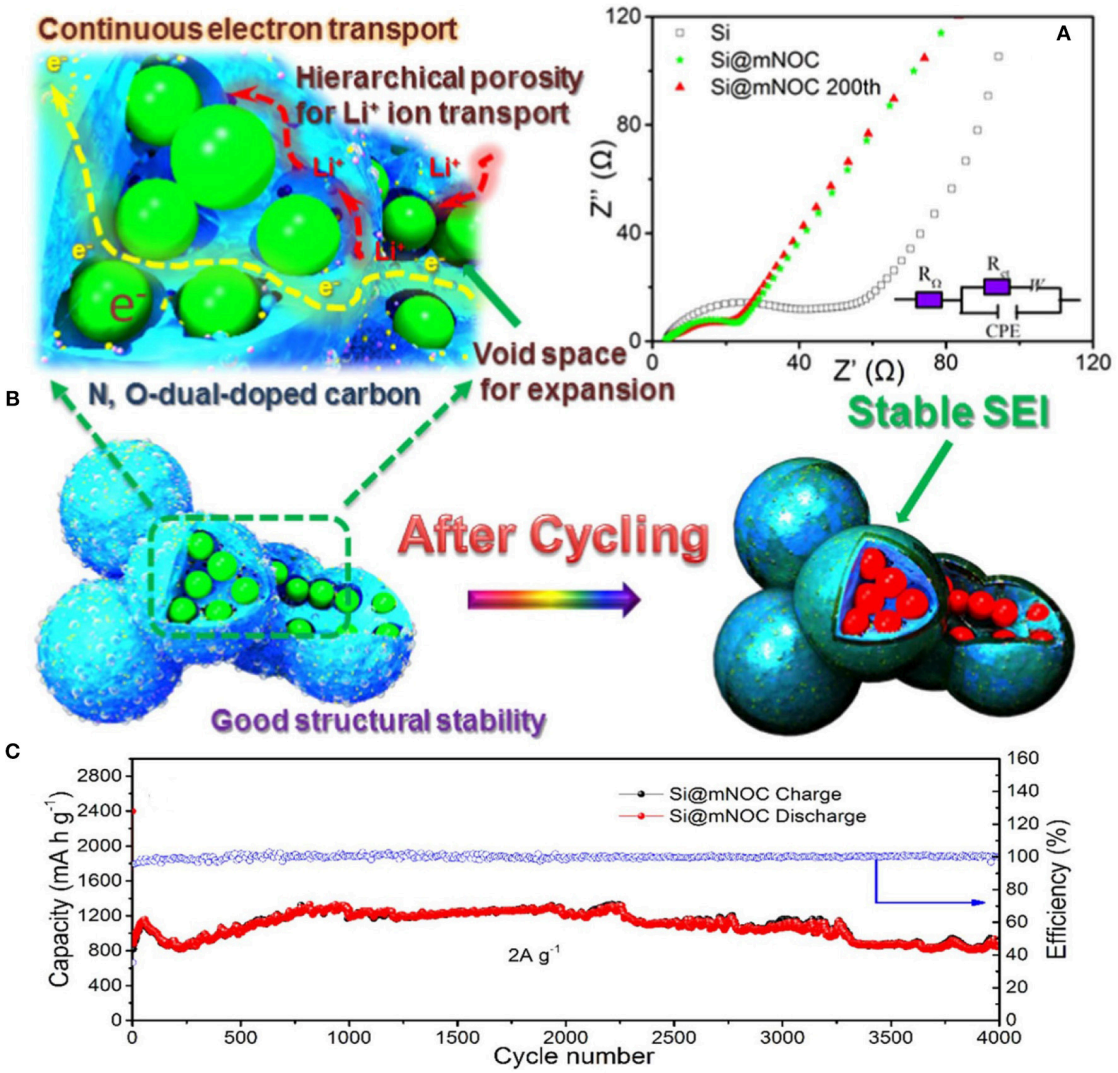
**(A)** Evaluation of SEI film and surface kinetics of Si@mNOC during cycling. **(B)** Schematic representation of conductivity, stability and stable SEI film on Si@mNOC after cycling. **(C)** Cycling performance with coulombic efficiency at current 2 A g^−1^. Reproduced with permission (Yu et al., 2016). Copyright © 2017 Elsevier Ltd. All rights reserved.

Continuous charging/discharging solid electrolyte interphase film by direct interaction between electrolyte and silicon particles is developed on silicon particles in the anode. SEI film on silicon particles acts as a barrier to further diffusion of lithium-ion and directly affects cycle life of Si anode (Yang et al., 2008; Zhou et al., 2014). Fast decay in capacity and SEI film has been controlled by a coating of different materials on silicon particles. Coating of different materials provides a barrier between direct contacts of electrolyte to active silicon which leads to decrease interfacial reaction between electrolytes to the electrode material and suppress the transformation of structure because of mechanical properties of coating materials (Kim et al., 2015). To stabilize SEI film, carbon has been used widely in the coating of silicon particles due to its good electronic and mechanical properties. Carbon coating controls the pulverization of silicon particles and prevents direct contact of the electrolyte with silicon particles (Yi et al., 2017). By leaving interior voids, unfilled pSiMPs was coated with carbon as shown in Figure 4. Unfilled carbon coated SiMPs as anode material delivered high capacity and excellent cycle life (1,500 mA h g^−1^ at current rate C/4 over 1,000 cycles). Exterior carbon coating prevented the direct contact electrolyte with silicon particles and interior voids provided extra space to silicon particles during volume expansion as in Figure 4 (Lu et al., 2015). Furthermore, Apple-like silicon@nitrogen, oxygen-doped carbon hierarchical mesoporous structure was prepared and used as anode material shown in Figure 5. Si@mNOC as anode material delivered good capacity and long cycle life (1,203 and 900 mA h g^−1^ at current rate 2 A g^−1^ after 2,000 and 4,000 cycles in Figure 5C). In Si@mNOC, the void space and mesoporous structure accommodated the volume expansion and facilitated ion transport and also controlled SEI film and improved the mechanical stability of the anode material. Nitrogen and oxygen doping improved the electronic conductivity and electrochemical performance of Si@mNOC anode shown in Figures 5A,B (Yu et al., 2016). Many attempts have been taken to enhance the kinetics of ionic diffusion and electrical conductivity of coating material (carbon) by doping of different atoms (oxygen, nitrogen, and sulfur) and by using hierarchical structure (Xu et al., 2015; Wang et al., 2016).

#### Yolk-shell and core-shell silicon nanostructures

**Figure 6:**
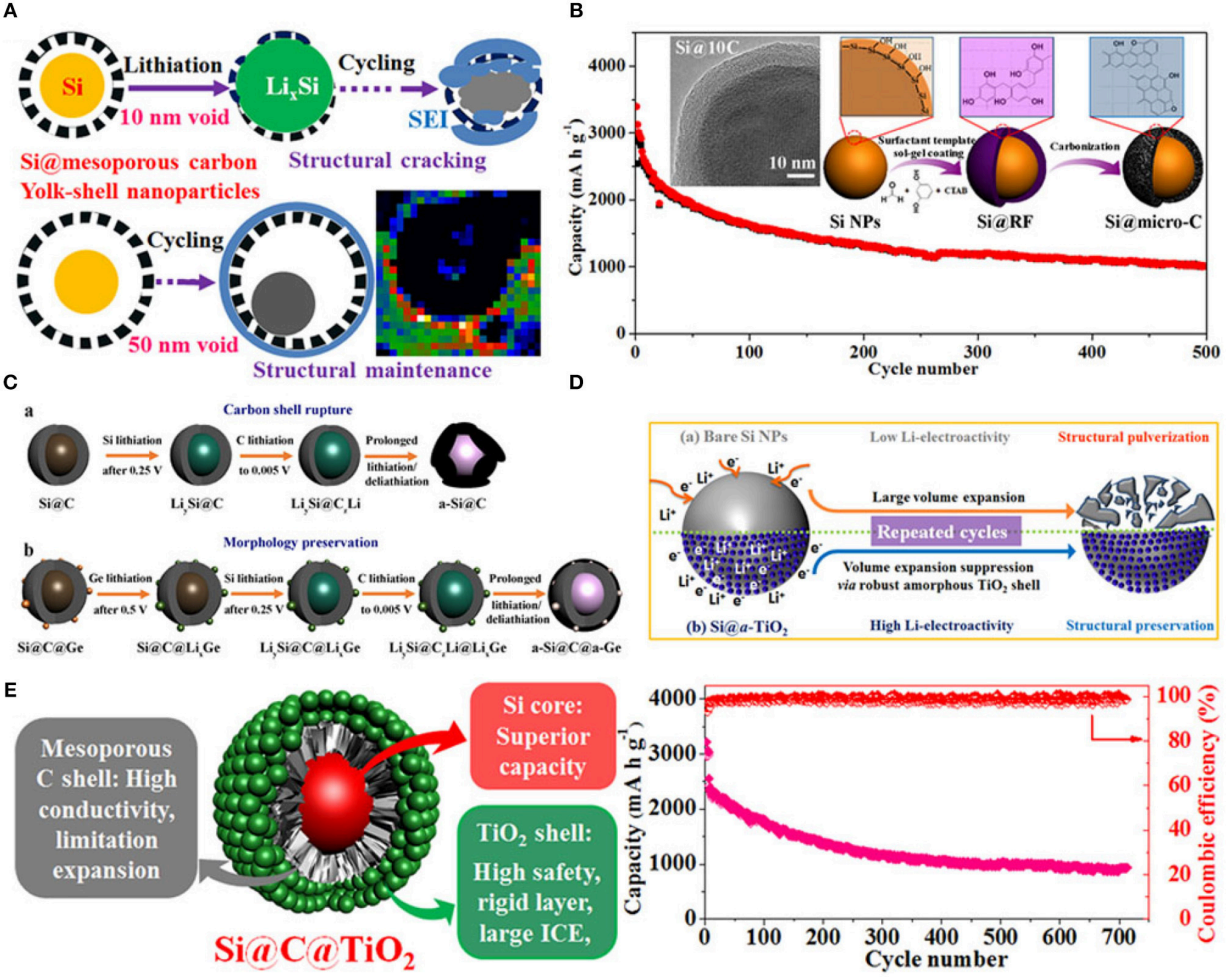
**(A)** Schematic representation of charging/discharging process of the yolk-shell structured Si@mC electrode with 10 and 50 nm voids. Reproduced with permission (Yang et al., 2015). Copyright © 2015 Published by Elsevier Ltd. **(B)** Schematic diagram of synthesis process of Si@micro-C composite, surface morphology, and cycle performance. Reproduced with permission (Luo et al., 2016b). © 2016 Elsevier Ltd. All rights reserved. **(C)** Schematic representation of charging/discharging process of Si@C and Si@C@Ge. Reproduced with permission (Luo et al., 2016a). **(D)** Schematic representation of the behavior of SiNPs and Si@TiO_2_ before and after cycling. Reproduced with permission (Yang et al., 2017). **(E)** Schematic illustration of conclusion and cycling performance of Si@C@TiO_2_ as anode material. Reproduced with permission (Luo et al., 2016c). Copyright © 2016, American Chemical Society.

Furthermore, electrochemical properties of silicon anode material enhanced by the modified structure of SiNPs and by using double coating of different materials (Ensafi et al., 2017; Jang et al., 2018; Xing et al., 2018). Herein, to improve electrochemical performance of anode material in LIBs, a series of surface and interface engineering to fabricate carbon and titanium oxide coated Si with yolk and core-shell structure have been fabricated as shown in Figure 6 (Luo et al., 2017). First, Si@mesoporous carbon yolk-shell structure was prepared which provided extra voids inside coating as shown in Figure 6A. This novel yolk-shell structure led to enhance rate capability, cycle life with good specific capacity and uniform stable SEI film which exhibited good electrochemical performance (Yang et al., 2015). Second, SiNPs coated with a controllable and uniform thickness of carbon coating (2–25 nm) to form a core-shell structure. This coaxial core-shell structure enhanced the stability of anode and exhibited good specific capacity over 500 cycles as shown in Figure 6B. (Luo et al., 2016b). Third, Si@C coated with germanium by a simple sol-gel process and formed Si@C@Ge core-satellite NPs as shown in Figure 6C. Coating of germanium on Si@C exhibited high electrochemical kinetic and good structural stability as compared to uncoated Si@C (Luo et al., 2016a). Fourth, to enhance initial coulombic efficiency, lithium storage safety, and structure integrity, amorphous TiO_2_ coated on SiNPs and made core-shell structure by the sol-gel process. Amorphous TiO_2_ shell provided high lithium storage safety by increasing lithium kinetic because of TiO_2_ showed a low resistance of Li diffusion during the electrochemical process as shown in Figure 6D. Amorphous TiO_2_ shell acted as an elastic belt on SiNPs to control volume variation during charging/discharging and stabilized the SEI film by resisting the contact of electrolyte to active silicon which has led to high cycle life (Yang et al., 2017). Last, SiNPs coated with double layer of carbon and TiO_2_ (Si@C@TiO_2_) via a two-step sol-gel process. Si@C@TiO_2_ composite as anode material handled conductivity, volume change during lithiation/delithiation and unstable SEI film. In Si@C@TiO_2_ composite carbon enhanced electrical conductivity by providing an electronic path to electron during the electrochemical process, TiO stabilized structure integrity of anode by providing mechanical properties and controlled the SEI film by stopping the direct contact of electrolyte to silicon as shown in Figure 6E (Luo et al., 2016c).

### Sensors

**Figure 7:**
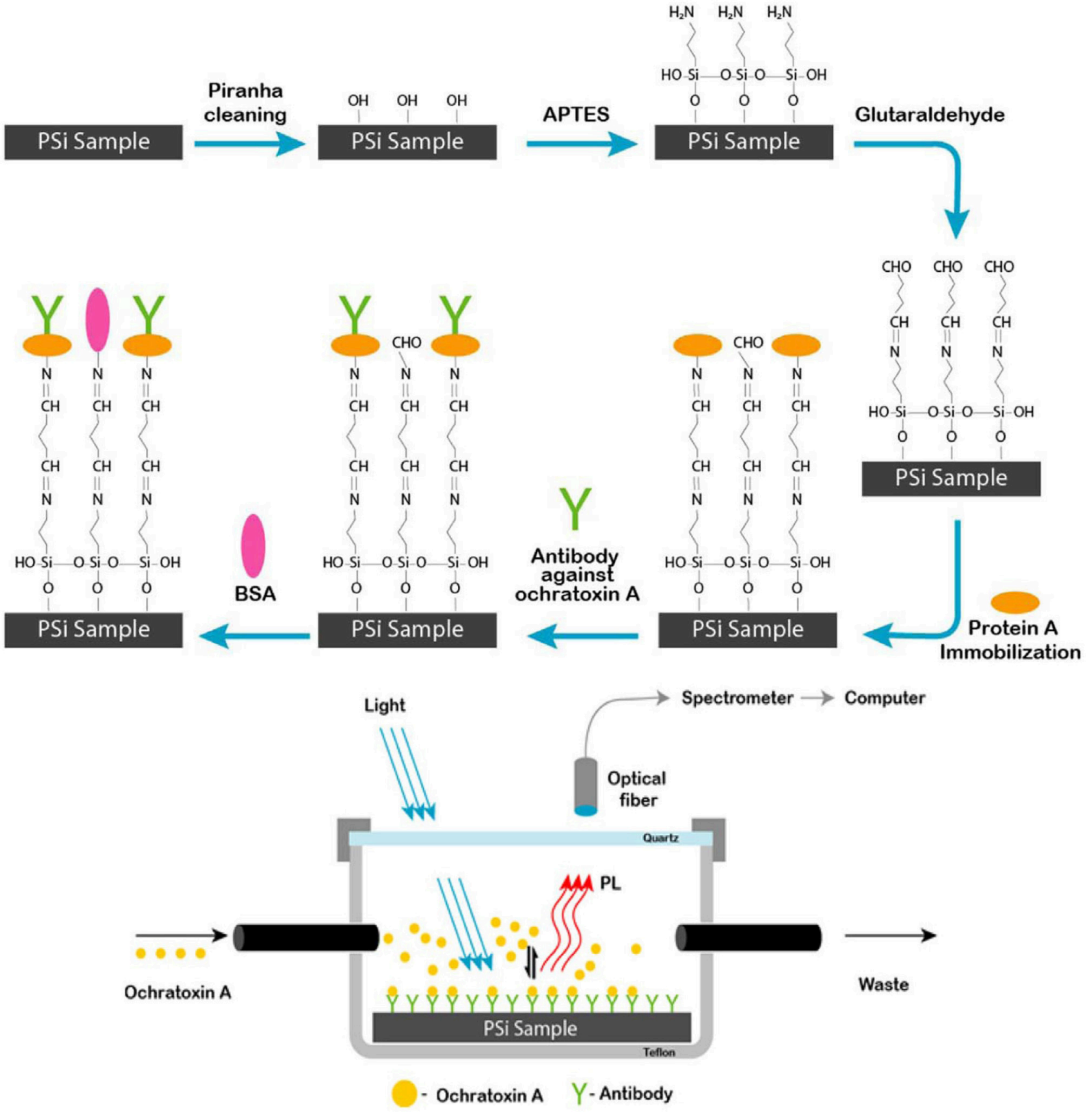
Schematic diagram of a process of synthesis porous silicon sample and setup for range of ochratoxin (0.01–5 ng/ml) detection by using pSi immunosensor under UV Laser. Protein A covalently immobilized surface on pSiNPs further modified by anti-OTA and BSA which was sensitive toward ochratoxin (Myndrul et al., 2017). Copyright © 2017 Elsevier B.V. All rights reserved.

**Figure 8:**
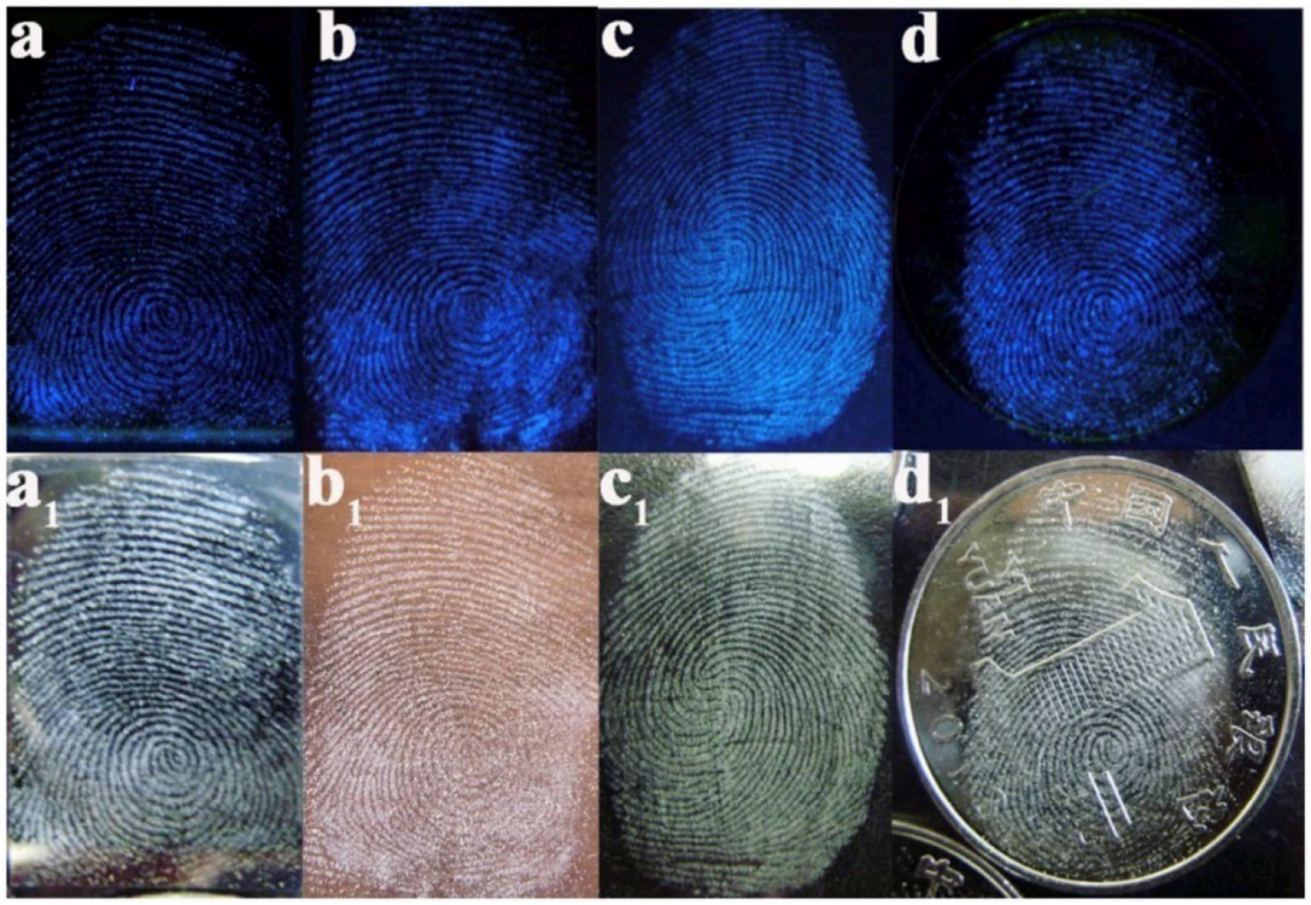
Latent fingerprint by SiNPs on a different sheet. **(a)** Sheet of the silicon wafer, **(b)** sheet of copper, **(c)** sheet of glass, **(d)** On patterned coin (Zhu et al., 2018). Copyright © 2017 Elsevier Ltd. All rights reserved.

On the basis of the surface, optical and electrical properties, pSiNPs have been used for detection of different atoms, gas molecules, pH and polar/non-polar organic solvent (Harraz et al., 2014; Kashyout et al., 2015; Sarkar et al., 2018). To improve the fluorescence properties of silicon oxides, these were coated with dye molecules through different functional groups. Photoluminescence intensity depends upon concentration and size of silicon particles. The doping of different particles also affected by other organic vapors which are under examinaion (Zhang et al., 2010; Huang et al., 2015; Moret et al., 2016; Nayef and Khudhair, 2018). On the basis of fluorescence quenching of pSiNPs with other atoms, pSiNPs have been used for detection of different ions and molecules (Cu^+2^, NO_2_, Hg^2+^, NH_3_, Ag^2+^, and ethyl carbamate) (Xia et al., 2013; Luo et al., 2018; Qin et al., 2018a,b). PL immunosensor was prepared by functionalization of porous silicon with Protein-A and bovine serum albumin (BSA) for detection of Ochratoxin A under UV Laser as shown in Figure 7. BSA added to active sites to blocking adsorption of protein on these sites and improve sensitivity. Functionalized-pSi immunosenor was tested under the wide range of concentrations (0.01–5 ng/ml) which was exhibited high sensitivity and high-speed detection even at low level of ochratoxin A. It was observed PL-intensity decreased as concentration of Ochratoxin A increased in sample (Myndrul et al., 2017). Carbon doped silicon nanoparticles were prepared by the mild reaction and used for detection Hg^2+^, Ag^2+^, and latent fingerprints. SiNPs showed good sensing ability for Hg^2+^ and Ag^2+^ by highly quenched with Hg^2+^, Ag^2+^ and provided high range wavelength of excitation and emission. SiNPs also used as fluorescence label to detect a fingerprint on different non-porous material surfaces. In the presence of ultraviolet excitation, SiNPs provided excellent fluorescent images on different surfaces as shown in Figure 8 (Zhu et al., 2018). SiNPs are easy to synthesize and due to numbers of properties (non-toxicity, wide range fluorescence spectra, good photoluminescence peak, solubility and large surface area) remained in great interest to apply in different fields.

### Other applications

**Figure 9:**
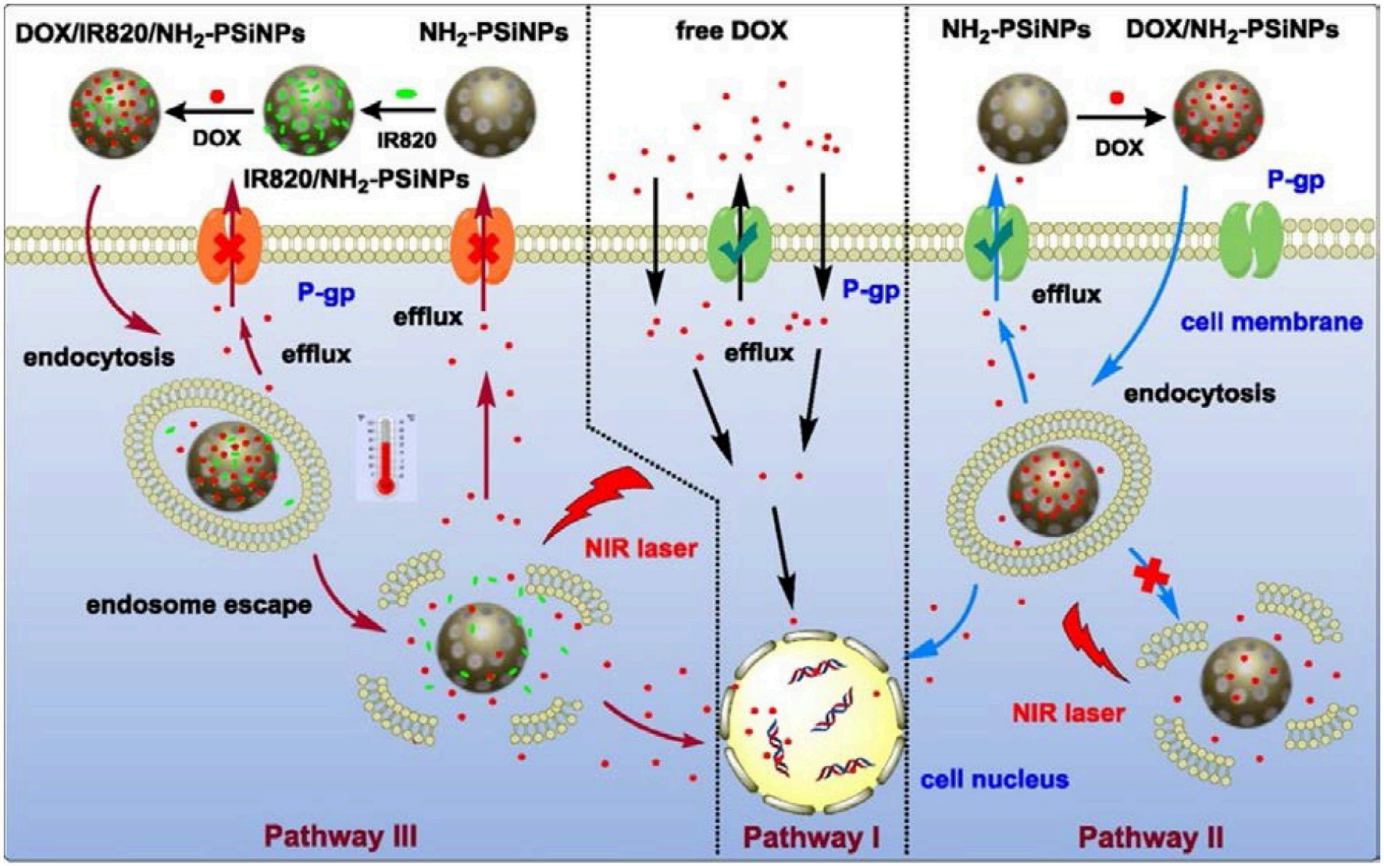
Schematic diagram of pSiNPs based composite and process of the pathway of DOX into nuclei of MDR cancer cell with pSiNPs and without pSiNPs and with pSiNPs and dye. In pathway I, free DOX without pSiNPs carriers injected in to MDR cancer cell and efflux of DOX molecules from cell was maximum. In pathway II, DOX with pSiNPs carriers into MDR cancer cell. DOX with pSiNPs killed the cancer cell under effect of NIR Laser and efflux of DOX molecules from cell is minimum. In pathway III, DOX with dye and pSiNPs entered to MDR cancer cell and killed the cancer cells completely under effect of NIR Laser without any efflux of DOX molecules (Xia et al., 2018). Copyright © 2018 Elsevier B.V. All rights reserved.

**Figure 10:**
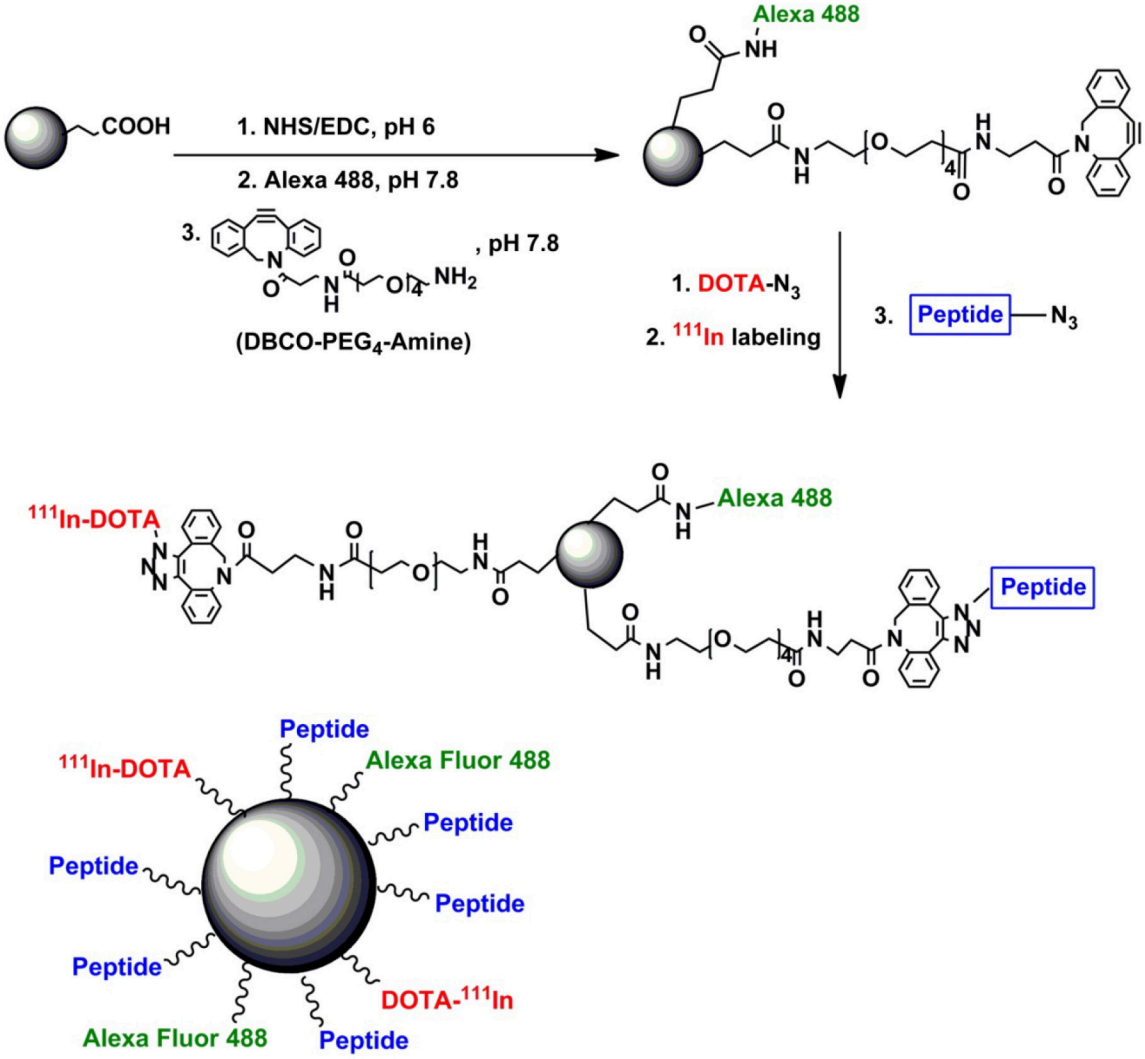
Schematic diagram of synthesis process of multifunctional mesoporous as nanocarrier. iRGD Peptide covalently attached with pSiNPs to achieve and suppress target cancer cell. Alexa flour 488 dye bonded covalently to pSiNPs, to monitoring distribution particles in cancer cell and DOTA introduced on pSiNPs to increase hydrophilic moieties (Wang et al., 2015). Copyright © 2015 Elsevier Ltd. All rights reserved.

On the basis of the surface, optical, biocompatible and nontoxic properties, pSiNPs have been used in bio-applications as Nano carriers, diagnostics and for the treatment of cancer (Vaccari et al., 2006; Donnorso et al., 2012; Haidary et al., 2012; Kaasalainen et al., 2012; Tzur-Balter et al., 2013; Yang et al., 2014b; Min-Dianey et al., 2018). The mSiNPs have great potential toward drug delivery devices due to their easy functionalization, good loading/release rate, solubility, tunable porosity and a large surface area (200–800 m^2^/g) (Reffitt et al., 1999; Anderson et al., 2003; Mattei and Valentini, 2003; Horcajada et al., 2004; Salonen et al., 2005; Anglin et al., 2008; Tabasi et al., 2012; Ma et al., 2017a). In bio-applications, pSiNPs exhibit good result *in vitro* and *in vivo* conditions (Ferreira et al., 2016). The pSiNPs were prepared by electrochemical etching and coated with dextran. The pSiNPs used for theranostics of cancer on the basis of photoluminescence properties and noted that excellent uptake of pSiNPs by cancer cell and also suppress the proliferation of cancer cells *in vitro* (Wang et al., 2012). SiNPs have been used for detection of microRNAs which acts as biomarkers of various diseases. The concentration of miRNAs was measured by a decrease of SiNPs fluorescence (Ding et al., 2018). Further, pSiNPs loaded with anticancer drugs and photo thermal agent by electrostatic assembly technique to treat the multidrug resistant cancer cells as shown in Figure 9. It is observed that pSiNPs leave the anticancer medicines 88.1% under different conditions and kill the multidrug resistant cancer cell. The pSiNPs as Nano carriers increased the efficiency of photo thermal therapy and chemotherapy (Xia et al., 2018). Furthermore, multifunctional pSiNPs were prepared by SPAAS click chemistry as shown in Figure 10. Multifunctional pSiNPs improved the rate of dissolution and cancer therapy. Uptake of multifunctional pSiNPs by tumors was enhanced due to the presence of iRGD peptide on the surface of pSiNPs and retained in tumors which suppressed tumors to further growth. Multifunctional pSiNPs exhibited well *in vivo* behavior and highest efficiency of drug delivery (Wang et al., 2015).

## Conclusion and outlook

In this review, we focus on the importance of pSiNPs with different structures in various fields. Silicon has much attractive material as an anode in LIBs due to their theoretical capacity (4,200 mA h g^−1^), intercalated and electrical properties but after some cycles of charging/discharging volume of silicon changes. Continuous volume changes in silicon anode lead to fractures and affect the electrochemical properties. Solid electrolyte interphase developed on the particle by an electrolyte which leads to electrical contact loss. These challenges pulverization, electrical loss and stable SEI film by mesoporous silicon have been elaborated. It is observed that SiNPs play an important role to control volume changes in Si-anode as compare to silicon bulk. To control pulverization in Si anode further structure of silicon particles has been modified to mesoporous, 3D and hollow spheres. By structural modification of SiNPs, a significant enhancement of electrochemical properties have been noted. Furthermore, to control volume change and improve electrical contact loss and stabilization of SEI film, SiNPs were coated with different materials (carbon, polymers other metals). Coating of different materials on SiNPs has significantly improved electrochemical properties and stabilize SEI films on particles. It is noted that the double coating of different material on particles enhanced electronic conductivity, capacity and control SEI film by stopping direct contact of electrolyte to particles. For future work, silicon anode material for commercial use still needs to be improved in volume change, stabilization of SEI film, high capacity, long cycle life, initial Coulombic efficiency and rate capability.

In biosensors and gas sensors, on the basis of optical and surface properties of pSiNPs have been used and exhibited good results. In sensors, porous silicon provides a number of void spaces to adsorption of different molecules, gases, drugs, and biomolecules. Structural dependent properties (optical, electrical and electrical properties) varies after adsorption of different molecules on the surface of porous silicon. This variation in properties are used to detect different materials. Further pSiNPs can be used to detect different compounds and biomarkers of different diseases which are still undetected.

Mesoporous structure of silicon provides a large surface area (200–800 m^2^ g^−1^), tunable optical properties and void spaces which act as good drug carriers. MSiNPs have been used as drug carriers, for therapy and detection of cancer cells due to their unique properties optical, non-toxicity, biocompatibility and surface properties. Detection and treatment of cancer tissues, tumors, and some biomarkers have been observed successfully by use of pSiNPs.

## Author contributions

All authors listed have made a substantial, direct and intellectual contribution to the work, and approved it for publication.

### Conflict of interest statement

The authors declare that the research was conducted in the absence of any commercial or financial relationships that could be construed as a potential conflict of interest.
